# Rethinking Out-of-School Tutoring: Engagement Pathways and the Uneven Impact on Students’ Holistic Competencies

**DOI:** 10.3390/jintelligence14040061

**Published:** 2026-04-08

**Authors:** Hui Yan, Han Xiao, Jianlin Yuan

**Affiliations:** Institute of Educational Sciences, Hunan University, Changsha 410082, China

**Keywords:** out-of-school tutoring, tutoring engagement, differentiated effectiveness, holistic competencies

## Abstract

Out-of-school tutoring, as a form of privatized compensatory education beyond formal schooling, has become increasingly prevalent, yet its role in fostering students’ holistic competencies remains insufficiently examined. Drawing on a student engagement perspective, this study investigates how different types of out-of-school tutoring, including academic, arts, and sports tutoring, are associated with the development of students’ holistic competencies. Data were drawn from a survey of 704 Grade 10 students in central China. Tutoring engagement during junior secondary school was measured using a self-developed Likert-scale instrument, while holistic competencies were obtained from official Comprehensive Quality Assessment records. The findings reveal differentiated effects across tutoring types. Academic tutoring shows no significant association with academic performance or other dimensions of holistic competence. In contrast, sports tutoring is positively associated with physical and mental health, and arts tutoring demonstrates a significant positive relationship with artistic literacy. Regarding engagement characteristics, simply increasing the number of programs or financial investment yields limited benefits. Instead, time investment and cognitive involvement in sports tutoring, as well as affective involvement in arts tutoring, are positively related to specific dimensions of holistic competence. These results suggest that the effectiveness of out-of-school tutoring depends less on participation amount and more on the nature of students’ engagement. The study highlights the uneven developmental returns of compensatory education and calls for a more balanced and development-oriented approach to tutoring participation.

## 1. Introduction

Modern education systems are primarily structured around formal schooling and supplemented by various forms of compensatory education (Bray, 2016). As educational stratification intensifies, parents and students are increasingly exhibiting diverse and differentiated educational demands. This differentiation has contributed to the rapid expansion of the out-of-school tutoring market, which has become a global phenomenon. Existing research has primarily examined private tutoring with a particular emphasis on its role in enhancing academic performance (Bray & Kwok, 2003; Berberoglu & Tansel, 2014). In practice, out-of-school tutoring can be broadly categorized into academic and non-academic forms. Academic tutoring, often conceptualized as shadow education (Bray, 1999) or private tutoring, closely replicates and reinforces the content of school curricula associated with high-stakes examinations. In contrast, non-academic tutoring refers to fee-based educational activities oriented toward personal interests and skills—such as painting, dance, piano, and basketball—whose content lies outside the scope of standardized entrance exams. This integrated educational perspective, which emphasizes both academic and non-academic aspects of student development, is referred to in the scholarly literature as holistic competencies (Chan & Yeung, 2019) or personal qualities (Duckworth & Yeager, 2015). In the Chinese academic and policy discourse, the concept is often captured by the term holistic suzhi (Luo & Chan, 2023). To ensure consistency in international scholarly communication, this study adopts the term holistic competencies (Chan & Yeung, 2019) throughout.

The theory of educational reproduction provides a framework for understanding the rise of compensatory education (Bae & Choi, 2024). Families’ investment in such education is often driven by a dual set of expectations: first, to improve academic performance through subject-based tutoring (Feldman & Matjasko, 2005); and second, to enhance skills and competencies in areas such as esthetics, physical fitness, and mental well-being through participation in arts and sports programs (M. Tan et al., 2021). These investments are often intended to gain advantages in both high-stakes entrance examinations and future employment opportunities, thereby reinforcing the intergenerational transmission of economic and cultural capital. In the explanatory chain of compensatory education, academic and non-academic skill enhancement, university admission, and future employment, the effectiveness of compensatory education in promoting both types of outcomes is the most foundational element. While prior studies have primarily examined the impact of subject-based tutoring on academic achievement (Cooper et al., 2006; Atalmis et al., 2016), the differentiated mechanisms through which non-academic programs contribute to students’ aesthetic, physical, and psychological development remain unclear. Moreover, there is a notable lack of research that integrates both academic and non-academic tutoring into a unified analytical framework. Addressing this gap requires adopting a holistic perspective to uncover the broader patterns through which out-of-school tutoring influences students’ holistic competencies, thereby providing foundational empirical support for the theory of educational reproduction.

Drawing upon the theory of learning engagement, the present study examines the impact of three types of out-of-school tutoring (i.e., academic, arts, and sports tutoring) on students’ holistic competencies through three dimensions of engagement: behavioral, cognitive, and affective, as well as engagement intensity. Such a nuanced and well-rounded analysis facilitates a more comprehensive understanding of how both the type of tutoring and the nature of engagement shape students’ holistic competencies. Finally, the study explores the implications of these findings for educational reproduction, offering practical guidance to parents and students in navigating tutoring choices, and providing empirical evidence to inform policy initiatives aimed at reducing academic burdens and promoting educational equity.

## 2. Literature Review

### 2.1. The Nature and Scope of Out-of-School Tutoring

As academic performance serves as a key determinant in selective entrance examinations, both students and parents tend to invest more heavily in subject-based tutoring. Consequently, this form of tutoring attracts wider participation, supports a larger market, and garners greater attention in educational research (Sun et al., 2020). Multiple overlapping terms have emerged to describe this phenomenon, including private supplementary tutoring (Bray & Kwok, 2003), shadow education (Bray, 1999), private tutoring, and extracurricular tutoring (Heyne et al., 2024). These forms of tutoring are commonly characterized by privateness, supplementation, and an academic orientation (Bray & Kwok, 2003; Kim & Lee, 2010), all of which fundamentally point to subject-based private tutoring that operates outside the formal school system. In addition, a broader concept, namely, extracurricular activities, has been proposed to encompass both academic and non-academic domains (Feldman & Matjasko, 2005; Shulruf & Wang, 2013). However, this term is highly inclusive, referring not only to fee-based tutoring provided by private institutions or delivered at home, but also to free or publicly funded learning opportunities provided by schools, community organizations, and volunteers. In the Chinese context, there is increasing parental emphasis on children’s non-academic development, and a growing number of tutoring institutions offer structured programs in the arts and sports (Zhou & Suntrayuth, 2025). Particularly with the promotion of holistic competency evaluation reforms (Luo & Chan, 2023), participation in non-academic tutoring, which focuses on esthetics, physical fitness, and well-being, has become common among primary and lower secondary students. In light of the comprehensive scope of extracurricular activities and the privatized nature emphasized in the concept of tutoring, this study proposes the notion of extra-school tutoring. This concept is characterized by three key features: (1) comprehensiveness, referring to the inclusion of both subject-based tutoring and non-academic skill development in areas such as arts and sports; (2) privateness, defined as the provision of tutoring services, academic or non-academic, through paid arrangements; and (3) compensatoriness, indicating that such learning takes place outside the formal school system as a supplementary form of education. This conceptual framework facilitates a more integrated analysis of how various forms of compensatory education beyond mainstream schooling shape students’ holistic competencies.

### 2.2. Measuring Student Engagement in Out-of-School Tutoring

Existing research offers limited comprehensiveness in measuring students’ participation in extra-school tutoring. Most studies rely on binary indicators (e.g., participation vs. non-participation) (He et al., 2021; Sun et al., 2020; Y. Zhang, 2018), financial expenditure (Bray, 1999), time investment (Zhan et al., 2013; Y. Zhang et al., 2021) and types of participation (Sun et al., 2020; Zhan et al., 2013) measure involvement in supplementary education. These approaches primarily emphasize quantitative intensity of participation, overlooking the qualitative dimensions of students’ learning experiences. From a learning theory perspective, participation in out-of-school tutoring is a learning activity that involves not only observable behaviors but also internal psychological processes such as motivation, emotion, and learning strategies. However, current measurement approaches seldom capture these affective and cognitive components, making them overly simplistic and in need of refinement.

As a form of learning, it is more appropriate to conceptualize students’ participation in out-of-school tutoring through the lens of student engagement. Student engagement refers to the time, energy, and effort students devote to their learning processes (Astin, 2014). Kuh emphasized that student engagement reflects the amount of time and energy students devote to educationally purposeful activities (Kuh, 2009). Likewise, Fredricks, Blumenfeld, and Paris conceptualized behavioral engagement as including students’ effort, persistence, and active participation in learning tasks, thereby incorporating learning intensity into the broader construct of engagement (Fredricks et al., 2004). Early research on engagement focused on formal schooling, emphasizing students’ psychological involvement and effort in school activities (Newmann, 1992), as well as their willingness to participate (Chapman, 2003). Research has shown that participation in school activities can reduce student alienation and enhance engagement (Newmann, 1981). Engagement is also closely linked to intrinsic motivation (Marôco et al., 2016). For instance, Connell’s Self-system Process Model highlights how contextual factors, namely, autonomy, interpersonal relationships, and a sense of belonging, influence engagement by meeting students’ basic psychological needs for competence, autonomy, and relatedness (Connell, 1990). Similarly, Finn (1989) noted that both behavioral and affective engagement play key roles in improving academic outcomes and reducing student alienation (Finn, 1989).

Over time, scholars have identified multiple dimensions of student engagement. Among the most widely recognized are behavioral, cognitive, and affective engagement (Finn & Zimmer, 2012). Behavioral engagement refers to students’ participation in school- and classroom-related activities, encompassing both basic learning behaviors and proactive or initiative-taking behaviors. Affective engagement refers to students’ affective responses to learning, including their sense of belonging and perceived value of educational experiences. Cognitive engagement involves the use of deep learning strategies, sustained effort, and self-regulation. Given the complex and multifaceted nature of out-of-school tutoring, a multidimensional engagement framework allows for a more nuanced and accurate measurement of student participation. Moving beyond surface-level metrics such as time or money spent (Zhan et al., 2013; Y. Zhang et al., 2021), this approach captures the depth of students’ behavioral, cognitive, and affective involvement in their out-of-school learning experiences.

### 2.3. Effectiveness of Out-of-School Tutoring and Educational Reproduction

Research on the developmental outcomes of out-of-school tutoring has primarily focused on academic tutoring and its effects on students’ academic achievement (Sun et al., 2020; Y. Zhang et al., 2021), though findings have been mixed. Some studies report significant gains in subjects such as Chinese, mathematics, and English (Y. Zhang & Xie, 2016), especially among rural students (Sun et al., 2020), and emphasize the benefits of peer tutoring (Alzahrani & Leko, 2018). Students often perceive tutoring as more effective than school instruction in exam preparation (Zhan et al., 2013). Timing also matters: summer tutoring appears to be more effective than term-time tutoring in improving mathematics performance (He et al., 2021). Conversely, other studies question the effectiveness of academic tutoring. Some report minimal or even negative effects (J. Tan, 2009), while others find no significant impact on academic performance (Y. Zhang et al., 2021). However, academic tutoring has also been associated with improved cognitive development, school engagement (Y. Zhang, 2018), and non-cognitive skill acquisition (Zhao, 2019).

Despite this growing body of research, the role of non-academic tutoring in promoting students’ holistic competencies remains underexplored. Rooted in the paradigm of whole-person development, promoting students’ holistic competence represents an aspirational goal, which challenges educational ideologies that prioritize mere knowledge transmission while neglecting students’ moral, social, and affective development (Luo & Chan, 2023). Holistic competencies encompass a broad range of skills (e.g., critical thinking, problem-solving), positive values, and attitudes (e.g., resilience, appreciation for others), which are essential for students’ lifelong learning and whole-person development (Chan & Yeung, 2019). In response to an overemphasis on test scores and entrance exam rates, the Ministry of Education of China launched a holistic competency evaluation reform in 2013, promoting multi-dimensional assessments that include moral character, academic skills, physical and mental health, artistic literacy, and social practice (Y. Zhang et al., 2018). This policy shift has encouraged growing parental interest in arts and sports programs as a means to cultivate non-academic strengths that may provide competitive advantages in both entrance exams and future employment. Nevertheless, empirical research remains limited on how participation in arts- and sports-related tutoring influences the development of these holistic competencies.

The motivations behind participation in tutoring can be further illuminated through the theory of educational reproduction. Since Pierre Bourdieu’s formulation of the theory of cultural reproduction (Bourdieu & Passeron, 1970/1990), the reproductive function of education has attracted sustained scholarly attention (Treiman & Yip, 1989; Treiman et al., 2003). Educational reproduction is understood as the process by which advantaged families maintain their social position by investing in compensatory educational activities outside the formal school system (Southgate, 2009). Families with greater economic and cultural capital are more likely to invest in frequent, high-quality tutoring, both academic and non-academic, aimed at enhancing their children’s competitiveness and securing access to elite educational pathways (Bray et al., 2014). This process facilitates the intergenerational transmission of privilege and consolidates social stratification.

Traditionally, educational reproduction has emphasized the role of academic tutoring in strengthening academic credentials. Within this framework, the effectiveness of tutoring is regarded as a prerequisite for the reproduction function: if tutoring enhances academic performance, reproduction is achieved; if not, the process is hindered. However, with increasing diversification in university admission criteria, academic achievement is no longer the only determinant of access to elite institutions. Many universities now consider students’ strengths in areas such as the arts, sports, and social engagement. This shift exposes limitations in the reproduction logic based solely on the effects of academic tutoring. At this point, it is critical to investigate how both academic and non-academic forms of out-of-school tutoring contribute to students’ holistic competencies. Such inquiry not only enhances our understanding of the educational value of tutoring but also provides a more comprehensive account of educational reproduction in contemporary contexts.

### 2.4. The Present Study

Based on the preceding literature review, three major gaps in the existing research can be identified. First, prior studies have predominantly concentrated on academic tutoring and shadow education, with limited attention given to non-academic forms of tutoring, such as programs in the arts, sports, and other developmental domains. Second, most research emphasizes the effects of academic tutoring on academic achievement, yet lacks integrative frameworks that simultaneously consider both academic and non-academic tutoring in relation to students’ holistic competencies. Third, the measurement of tutoring participation has been constrained to quantitative aspects, centered on frequency, duration, or financial cost, while failing to capture students’ cognitive, affective, and behavioral engagement in different tutoring contexts.

To address these gaps, the present study adopts a student engagement perspective to investigate how participation in various types of out-of-school tutoring influences students’ holistic competencies. This approach aims to provide a more comprehensive and nuanced understanding of how academic and non-academic tutoring affect students’ cognitive, psychological, and social development. Specifically, this study seeks to address the following four research questions:

1. What are the overall characteristics of students’ participation in out-of-school tutoring?
2. How does participation in different types of tutoring influence students’ holistic competencies?
3. How do different types of out-of-school tutoring engagement affect the development of holistic competencies?
4. How do the patterns of out-of-school tutoring’s effects on students’ holistic competence reshape our understanding of the reproduction function in compensatory education systems?

## 3. Methods

### 3.1. Theoretical Framework

This study adopts student engagement theory (Bray et al., 2014) as its primary theoretical framework, integrating established approaches for measuring student participation in private tutoring and out-of-school programs. In this research, student engagement in out-of-school tutoring is conceptualized along three key dimensions: cognitive engagement, affective engagement, and behavioral engagement, with engagement intensity incorporated as a key quantitative factor of tutoring involvement. Engagement intensity refers to the quantitative aspects of participation, including the number of tutoring programs attended, financial expenditure, and time invested. Cognitive engagement captures the strategies students employ during tutoring, such as their awareness of program content and difficulty, alignment with personal learning needs, self-regulation and monitoring during learning, and the enhancement of learning strategies. Affective engagement refers to students’ attitudes toward tutoring, including their autonomy in selecting programs, perceived learning efficacy, satisfaction with the instructional environment, and overall emotional experience. Behavioral engagement is indicated by observable actions such as regular attendance, classroom participation, task completion, and the attainment of learning objectives.

The theoretical framework of holistic competence is derived from the Ministry of Education of China’s policy on comprehensive quality evaluation. This framework comprises five key dimensions: moral character, academic performance, social practice, artistic literacy, and physical and mental health. The moral character dimension assesses students’ values and social development, including ideals and beliefs, integrity, empathy, sense of responsibility, and legal awareness. The academic performance dimension focuses on students’ construction of knowledge systems, encompassing theoretical understanding, skill application, and problem-solving abilities. The social practice dimension evaluates students’ engagement in social and experiential contexts, particularly through hands-on learning and community involvement. The artistic literacy dimension measures esthetic appreciation, artistic comprehension, interpretive skills, and creative expression. The physical and mental health dimension assesses overall well-being, including physical functioning, healthy lifestyle habits, motor skills, exercise routines, and psychological resilience.

By integrating the frameworks of student engagement and holistic competence, the study establishes a comprehensive analytical basis to examine how various forms of engagement in different types of out-of-school tutoring contribute to students’ multidimensional development.

### 3.2. Variables and Measurements

This study incorporates three categories of variables, as summarized in the Appendix A. The covariates include a range of student background characteristics that serve as control variables in the analysis. These consist of gender, household registration status, school type, parental education level, annual household income, and academic achievement.

The independent variables focus on students’ engagement in out-of-school tutoring, which is categorized into three types: academic tutoring, sports tutoring, and arts tutoring. Drawing on the student engagement framework (Bray et al., 2014), each tutoring category is assessed across three dimensions: behavioral engagement, cognitive engagement and affective engagement, with engagement intensity incorporated as a key quantitative factor for tutoring involvement.

Engagement intensity captures the quantitative aspects of tutoring participation and is measured using three observed indicators: the number of tutoring programs a student is involved in, the financial expenditure associated with these programs, and the amount of time invested in each category. Engagement intensity is treated as observed variables in the statistical models. In contrast, cognitive, affective, and behavioral engagement are treated as latent variables and are measured through multiple items developed for this study. Based on the theoretical framework proposed by Finn and Zimmer (2012), this study utilizes self-developed measurement instruments tailored to the out-of-school tutoring context. All items measuring latent variables were rated using a 5-point Likert scale (refer to Appendix C for the item list).

The dependent variables, namely, students’ holistic competence across the five dimensions, are measured using the Comprehensive Quality Assessment Project for Junior Secondary School Students, which is administered by educational authorities. This assessment follows the principles of performance-based assessment and adopts a portfolio approach, using a digital platform to document students’ performance in each of the five areas throughout their junior secondary education. Upon students’ graduation from lower secondary school, their holistic competence is jointly evaluated by education administrative authorities and schools in accordance with standardized rules and procedures. The assessment outcomes are classified into four levels (A, B, C, and D) and serve as an important reference for admission decisions in the high-stakes upper secondary entrance examination. As such, the evaluation carries substantial authority and public credibility, providing robust support for the reliability and validity of the assessment of students’ overall competencies.

### 3.3. Data Collection and Scale Reliability

Data for this study were collected through on-site questionnaire administration. The target participants were Grade 10 students who had just entered senior high school in a provincial capital city in central China. These students had recently completed the entrance examination for senior high school (Zhongkao), which allowed them to accurately recall their participation in out-of-school tutoring during junior secondary school, as well as their academic performance and holistic competence assessment outcomes. High school students in the sample were drawn from a wide range of lower secondary schools, which effectively ensured the representativeness of the research participants in terms of their lower secondary educational backgrounds. Although recall bias cannot be entirely ruled out, the study focused on structured activities, which are less susceptible to recall errors than transient or momentary interactions. To further reduce sensitivity to potential recall inaccuracies, participation intensity in this study was measured using ordinal categories rather than precise quantitative counts. A total of 800 questionnaires were distributed, and 704 valid responses were ultimately retained for analysis. Prior to data collection, this study obtained approval from the Institutional Ethics Committee and strictly adhered to the principles of informed consent. All participants took part voluntarily, and the questionnaire was administered anonymously to ensure participant privacy and information security. The data collected are used exclusively for academic research purposes. The study sample is composed of approximately 47.8% female students and 52.2% male students. Regarding household registration, approximately 65.1% of students hold urban household registration, while the remainder hold rural registration. The sample covers both key schools (55%) and non-key schools (45%), providing representative coverage of diverse junior secondary educational backgrounds. In addition, all control variables were carefully coded prior to analysis. In particular, primary school academic rankings were reverse-coded.

To ensure the robustness of the measurement instruments, the reliability and validity of the scales used to assess students’ engagement in out-of-school tutoring were empirically tested using the collected survey data. The internal consistency of the engagement dimensions was evaluated across the three tutoring types. The Cronbach’s alpha values for these dimensions ranged from 0.85 to 0.93, reflecting a high level of internal reliability and confirming that the scales were suitable for capturing the multidimensional nature of student engagement in various tutoring contexts.

To assess the construct validity of the engagement scales, a confirmatory factor analysis (CFA) was conducted, modeling nine latent factors representing behavioral, cognitive, and affective engagement across academic, arts, and sports tutoring. The model showed acceptable fit: RMSEA = 0.057, CFI = 0.887, TLI = 0.878, and SRMR = 0.048. These indices suggest an acceptable fit given the complexity of the multi-factor measurement model (Salum et al., 2012). All factor loadings ranged from 0.623 to 0.881 and were statistically significant at the *p* < 0.001 level. In addition, a CFA was conducted to examine the measurement structure of the Comprehensive Quality Assessment. The results indicated an acceptable model fit: RMSEA = 0.035, CFI = 0.995, TLI = 0.989, SRMR = 0.016). All factor loadings ranged from 0.459 to 0.738 and were statistically significant at the *p* < 0.001 level. In addition, the factor correlations ranged from 0.260 to 0.628, providing empirical support for the discriminant validity among the various dimensions. These results provide strong evidence for the structural validity of the engagement scales. In addition, multicollinearity among the key independent variables was examined. Variance inflation factors (VIFs) were calculated for all predictors, and the results showed that the maximum VIF was 1.90, which is well below the commonly used threshold of 10. This indicates no serious multicollinearity concerns.

### 3.4. Analytical Procedures

This study investigates how different types of out-of-school tutoring and their associated engagement dimensions affect students’ holistic competencies. As the goal is not to construct a complex causal model among independent variables, a multiple regression approach is employed.

The analysis follows three steps. First, descriptive statistics are used to summarize key variables and provide an overview of students’ tutoring participation and holistic competence levels, including variation across student background characteristics. Second, multiple regression models estimate the overall effects of academic, arts, and sports tutoring on holistic competence. This step identifies the distinct contributions of each tutoring type. Third, a more fine-grained set of regression models examines the effects of specific engagement dimensions within each tutoring category. Behavioral, cognitive, and affective engagement are represented by factor scores derived from confirmatory factor analysis, reducing measurement error in the regression models.

It should be noted that tutoring-specific engagement is only meaningful for students who actually participated in the corresponding tutoring type. Therefore, for students without sports, arts, or academic tutoring experience, engagement variables were initially coded as 0 in the dataset and subsequently treated as missing values in the regression analyses. As a result, these non-participants were excluded from the tutoring-specific engagement models via listwise deletion, ensuring that engagement effects were estimated only among relevant participants.

The specifications of these regression models are presented in the following sections:

(1) $$y_{i}=\beta_{0}+\beta_{1}{phy}_{i}+\beta_{2}{arts}_{i}+\beta_{3}{academ}_{i}+\beta_{m}{Cov}_{mi}{+\varepsilon }_{i}$$

Specifically, the models examine the effects of student engagement in sports, arts, and academic tutoring on each of the five dimensions of holistic competence. Accordingly, five separate regression models are estimated, each corresponding to a specific aspect of holistic competence. In these models, $y_{i}$ represents one of the five dimensions of holistic competence. The key predictors include the independent variable ${phy}_{i}$ (overall engagement in sports tutoring), ${arts}_{i}$ (overall engagement in arts tutoring), and ${academ}_{i}$ (overall engagement in academic tutoring). Each overall engagement score is calculated as the average of four components: engagement intensity, behavioral engagement, cognitive engagement, and affective engagement. ${Cov}_{mi}$ represents the nine covariates included in the study.

(2) $$y_{i}=\beta_{0}+\beta_{1}{amo}_{i}+\beta_{2}{mon}_{i}+\beta_{3}{tim}_{i}+\beta_{4}{beh}_{i}+\beta_{5}{con}_{i}+\beta_{6}{aff}_{i}+\beta_{m}{Cov}_{mi}{+\varepsilon }_{i}$$

The model examines the effect of different types of tutoring engagement on the five dimensions of students’ holistic competence for different subject types separately, again containing five models. The dependent variable $y_{i}$ is indicative of a dimension of student holistic competence, the independent variable ${amo}_{i}$ is the number of participation in the type of tutoring, ${mon}_{i}$ is the monetary cost of engaging in the type of tutoring, ${tim}_{i}$ is the time spent engaging in the type of program, ${beh}_{i}$ is the behavioral engagement of the type of program, ${con}_{i}$ is the cognitive engagement of the type of program, and ${aff}_{i}$ is the affective engagement, the model actually contains a total of 18 core independent variables for the sports, arts, and academic tutoring. ${Cov}_{mi}$ represents the nine covariates included in the study.

To reduce sensitivity to heteroskedasticity and to provide more robust statistical inference, this study reports bootstrap standard errors based on 5000 resampling replications. Statistical significance is therefore evaluated using these bootstrap-based estimates. All statistical analyses were conducted using SPSS 27.0.

## 4. Results

### 4.1. Overview of Out-of-School Tutoring Engagement and Students’ Holistic Competencies

Table 1 presents descriptive statistics on students’ out-of-school tutoring engagement and holistic competencies. Overall, 95.3% of students participated in at least one type of tutoring, with academic tutoring showing the highest rate (90.2%), followed by arts (67.5%) and sports (54.2%). In terms of engagement intensity, which is reflected in the number of programs attended, financial cost, and time investment, academic tutoring significantly surpassed the other two categories. This finding reaffirms the academically driven nature of compensatory education, as supported by existing research and public perception.

Students performed well across all five dimensions of holistic competence, with mean scores exceeding 3.7 on a four-point scale (A = 4, B = 3, C = 2, D = 1). Grade distribution analysis showed the highest proportion of “A” ratings in moral character (93.8%), followed by artistic literacy (83.3%), social practice (79%), physical and mental health (79%), and academic performance (71.6%).

### 4.2. Group Differences in Out-of-School Tutoring Engagement

To assess differences in tutoring engagement intensity across student subgroups, independent-samples t-tests or one-way ANOVA were conducted based on background variables. Appendix B summarizes the results, reporting only t-values or F-values and significance levels for brevity.

Significant gender differences emerged in arts and sports tutoring: male students participated more in sports programs, while female students engaged more in arts tutoring. No significant gender difference was found for academic tutoring. Urban students had significantly higher participation and financial investment in both sports and academic tutoring compared to rural students, though rural students spent more time on sports programs. Students from key junior secondary schools reported significantly more engagement in all three tutoring categories, including sports, arts, and academic tutoring, in terms of both program participation and financial cost. A strong correlation was also found with family socioeconomic status: students with more highly educated parents and higher household incomes were more involved in tutoring and invested more money across all categories. Aside from higher arts tutoring participation among students who attended primary school outside the local district, variables such as primary school academic ranking and location had limited impact on tutoring intensity.

Overall, students with urban household registration, key school backgrounds, and stronger family economic and cultural capital were significantly more engaged in out-of-school tutoring. These results highlight how structurally advantaged families are better positioned to invest in supplementary education resources.

### 4.3. Effects of Different Types of Out-of-School Tutoring on Holistic Competencies

Controlling for all relevant covariates, Model 1 assessed the impact of overall engagement in sports, arts, and academic tutoring on the five dimensions of students’ holistic competencies. Results from the five regression models are summarized in Table 2.

The analysis revealed that academic tutoring engagement did not have a significant effect on any dimension of holistic competencies. By contrast, sports tutoring was positively associated with physical and mental health (β = 0.17), while arts tutoring showed a significant positive effect on artistic literacy (β = 0.11). No other significant associations were identified between sports or arts tutoring and the remaining dimensions of holistic competence.

Among the covariates, students from higher-ranked junior secondary schools tended to perform better across various dimensions of holistic competence. Additionally, students whose fathers had higher levels of education scored better in academic performance.

Gender differences were also observed: male students outperformed female in academic performance, whereas female students showed higher levels of artistic literacy. Moreover, students who attended both primary and junior secondary schools within the same city demonstrated stronger outcomes in artistic literacy and physical and mental health.

### 4.4. Effects of Different Types of Tutoring Engagement on Holistic Competencies

This section presents the core findings of the study, focusing on how different dimensions of engagement in out-of-school tutoring affect students’ holistic competencies across sports, arts, and academic tutoring. Controlling for nine covariates, Model 2 estimates five separate regression models, each corresponding to one dimension of holistic competencies. Table 3 summarizes the results; coefficients for covariates are omitted for brevity.

In terms of engagement intensity, the number of tutoring programs attended showed no significant effect on any dimension of holistic competence. This indicates that simply participating in more tutoring programs does not necessarily lead to better developmental outcomes. Similarly, financial investment in sports and arts tutoring was not significantly associated with any outcomes. However, financial investment in academic tutoring showed a positive effect on students’ moral character. This may reflect the evaluative criteria for moral character, which often include participation in community service, leadership, and charitable activities. Such opportunities are generally more accessible to students from higher-income families, who are also more likely to invest more in tutoring. Time investment in arts tutoring had no significant effect on any competence dimension. In contrast, time spent on sports tutoring was positively associated with students’ social practice and physical and mental health. On the other hand, time spent on academic tutoring had significant negative effects on artistic literacy (β = −0.14) and physical and mental health (β = −0.19), suggesting that excessive academic tutoring may detract from students’ esthetic development and well-being.

Among the non-intensity dimensions, cognitive engagement in sports tutoring had a positive impact on social practice (β = 0.23), supporting the notion that meaningful participation in sports enhances real-world application skills, while this pattern may partly result from unmeasured confounding factors. Moreover, both time investment and cognitive engagement in sports contributed positively to social practice. Affective engagement in arts tutoring was strongly associated with moral character (β = 0.52), highlighting the importance of affective involvement in fostering artistic growth.

Overall, the findings emphasize that the quality and nature of engagement, particularly cognitive and affective components, play a more critical role in shaping students’ holistic competencies than the mere intensity or frequency of participation.

## 5. Discussion

**RQ1:**  *Student participation in out-of-school tutoring is extremely high and heavily concentrated in academic subjects. Engagement levels are significantly higher among students from families with greater economic and cultural capital.*

Survey findings reveal that out-of-school tutoring is nearly universal, with only 4.7% of students reporting no participation. Academic tutoring showed the highest participation rate at 90.2%, followed by arts (67.5%) and sports (54.2%) tutoring. Across all dimensions of engagement—program count, financial investment, and time spent—academic tutoring consistently surpassed the other categories, reinforcing previous research that compensatory education in China remains strongly academically driven (W. Zhang & Bray, 2021).

Group-level comparisons highlight pronounced disparities in tutoring engagement based on socioeconomic background. Students with urban household registration, more educated parents, higher household incomes, and those enrolled in key junior secondary schools were significantly more likely to engage in all three types of tutoring and to invest more time and financial resources. These patterns underscore persistent inequalities in access to supplemental education and affirm the powerful influence of family capital in shaping students’ learning opportunities beyond the formal school system.

**RQ2:**  *The effects of different types of out-of-school tutoring on students’ holistic competencies are distinct.*

Educational expectations strongly shape the nature of tutoring participation. Families and students generally expect academic tutoring to enhance academic achievement (Y. Zhang et al., 2021; Y. Zhang & Xie, 2016), while arts and sports programs are seen as pathways to improving artistic literacy and physical and mental well-being. These expectations reflect a broader differentiation in the perceived purposes of education. The findings confirm that the effects of tutoring vary by type, revealing a clear pattern of differentiated utility. Specifically, sports tutoring is positively associated with students’ physical and mental health, and arts tutoring significantly correlates with artistic literacy. These results align with common assumptions that physical activity promotes fitness and health, while artistic engagement fosters esthetic and expressive capacities.

In contrast, academic tutoring did not show a significant effect on students’ academic performance within the holistic competence framework, which contradicts common expectations. Although numerous studies have demonstrated the positive impact of academic tutoring on achievement (Berberoglu & Tansel, 2014), there is also a body of research that finds limited or no significant effects (Y. Zhang et al., 2021). This inconsistency likely arises from the complex factors influencing student development. Studies lacking a comprehensive set of explanatory variables may yield inconclusive findings. By examining multiple tutoring types, integrating a multidimensional engagement model, and assessing five distinct dimensions of holistic competence, this study provides a more nuanced understanding of how different types of tutoring contributes to overall student development.

**RQ3:**  *The effects of different types of engagement in out-of-school tutoring on students’ holistic competencies are differentiated.*

First, the number of tutoring courses in which students participate is not significantly associated with overall competence development. In fact, greater time investment in academic tutoring is associated with significant negative effects on students’ artistic literacy and physical and mental well-being. While this pattern may partly result from unmeasured confounding factors, it nonetheless challenges the conventional belief that “more tutoring inevitably leads to better outcomes.” Simply increasing the number of tutoring programs does not guarantee substantive educational gains. Excessive academic tutoring may undermine students’ well-being and creativity, likely due to heightened pressure, reduced leisure time, and an overemphasis on academic pursuits at the expense of other domains of development (Cheng & Huang, 2022).

Second, both time investment and cognitive engagement in sports tutoring positively influence students’ physical and mental health as well as social practice. Although the number of sports programs attended shows no significant effect, the quality of involvement through sustained time and cognitive effort proves beneficial. These results support the widely accepted view that consistent, mentally engaged physical activity promotes fitness, well-being, and practical skills, highlighting the importance of long-term, focused participation in sports.

Third, affective engagement in arts tutoring significantly enhances artistic literacy, while other factors such as program count, time, and financial investment show no notable effects. This underscores the affective and intrinsic nature of artistic development, rooted in personal expression and esthetic experience. Cultivating artistic literacy depends more on students’ emotional connection and motivation than on the amount of participation. Encouraging students to engage in art forms that resonate personally may be more effective for artistic growth than simply increasing external inputs.

**RQ4:**  *The Reproductive Function of Compensatory Education Is Limited.*

Out-of-school tutoring involves complex issues of social reproduction, educational competition, equity, and student well-being. This study provides a clearer understanding of the mechanisms and limits of compensatory education within the context of educational reproduction. The positive effects of targeted educational interventions on fostering students’ 21st-century competencies and career aspirations have been empirically verified in existing research, which also highlights the long-term holistic developmental value of well-designed extracurricular and specialized educational programs (Mahmood et al., 2025).

First, the effectiveness of compensatory education exhibits a differentiated pattern. By incorporating academic tutoring, arts programs, and sports programs into a single analytical model, this study examines the effects of different types of out-of-school tutoring and different forms of investment—namely investment intensity, behavioral engagement, cognitive engagement, and emotional engagement. The results indicate clear differentiation in the effectiveness of compensatory education.

On the one hand, there are pronounced differences across tutoring types. Investment in sports programs is positively associated with students’ physical and mental health, and participation in arts programs is positively related to the development of artistic literacy, whereas academic tutoring shows no substantive association with improvements in academic achievement.

On the other hand, differentiation is also evident across types of investment. The number of out-of-school tutoring programs in which students participate is not significantly associated with any dimension of holistic competence. In contrast, time and cognitive investment in sports programs are positively related to the development of social practice competencies and physical and mental health, while emotional investment in arts programs is positively associated with artistic literacy.

Within the East Asian educational context, participation in a greater number of out-of-school tutoring activities is commonly expected to facilitate students’ multidimensional development (Bray & Lykins, 2012). From the perspective of learning effectiveness, however, the findings of this study diverge from the conventional belief in “high-intensity” academic tutoring and instead affirm the positive value of engagement in sports programs and arts programs in which students are actively and intrinsically motivated.

Second, the reproductive function of compensatory education appears limited. According to educational reproduction theory (Treiman & Yip, 1989; Treiman et al., 2003), families with greater economic and cultural capital access more and better-quality tutoring to boost academic performance, facilitating entry to elite schools and high-status careers. Academic achievement is the key selection criterion, and academic tutoring’s effectiveness is critical for this reproduction mechanism. However, this study’s findings indicate academic tutoring does not significantly improve academic performance, challenging this traditional logic. While sports and arts tutoring contribute to well-being and artistic growth, these dimensions carry less weight in selective admissions and social competition compared to academic performance (Bray, 2012). Thus, although beneficial developmentally, non-academic tutoring has limited power in reproducing social advantage.

## 6. Implications

Reducing students’ academic burden and regulating out-of-school tutoring have become shared policy concerns worldwide, especially in East Asian countries where intense academic competition and widespread supplementary tutoring are common (OECD, 2019). Although targeted policies—such as the “Double Reduction” policy in China (Xue & Li, 2023), the Act on the Regulation of Teaching Outside Regular Schools in Korea (Ministry of Education, Korea, 2006), “Teach Less, Learn More” initiative in Singapore, the Child Welfare Act in Japan (Roesgaard, 2006), and the Directive No. 27 in Vietnam (Prime Minister of Vietnam, 2021)—have been introduced to alleviate academic pressure and regulate tutoring, the culture of private tutoring remains deeply ingrained. Despite these formal restrictions, 83% of East Asian parents still view tutoring as a necessary investment to maintain educational advantage (OECD, 2019), raising doubts about the effectiveness of policy regulation. The core challenge lies in the deeply rooted belief in the compensatory function of education, which fuels strong, self-driven demand. Parents and students often assume that “the more one studies, the better the outcomes”, leading many to bypass restrictions through informal or disguised tutoring. This tendency may compromise students’ well-being and limit opportunities for holistic competencies in pursuit of academic gains. This study contributes to reframing the discourse on out-of-school tutoring by providing empirical evidence on the differentiated impacts of academic, arts, and sports tutoring on students’ holistic competencies. The findings offer valuable insights to help redefine family perceptions of tutoring and inform future policies aimed at reducing academic pressure and better regulating private tutoring.

First, parents and students should recognize the differentiated effectiveness of different types of tutoring and reduce their heavy reliance on academic tutoring, while considering moderate participation in non-academic programs. Consistent with recent studies (Y. Zhang et al., 2021), this study shows no substantive association between academic tutoring and improvements in academic achievement. Increased time and financial investment in academic tutoring may therefore not deliver the expected improvements. Instead, time and cognitive engagement in sports and affective engagement in the arts are positively associated with student development in health and artistic literacy. Participation in non-academic activities aligned with students’ interests and developmental needs may thus be more beneficial.

Second, when choosing sports or arts tutoring programs, students and parents should prioritize alignment with individual needs and focus on enhancing cognitive and affective engagement. Neither the number of programs nor financial investment in non-academic tutoring significantly affects holistic competencies. This implies that participation should not be driven by quantity or cost, but by matching program content to personal interests and developmental goals. Cognitive engagement, including thoughtful learning strategies and goal-oriented self-regulation, is especially important for fostering artistic literacy and physical and mental well-being.

Third, the underlying principles of recent compensatory education governance policies are scientifically supported. Across East Asia, policy reforms commonly aim to reduce reliance on academic tutoring, encourage moderate participation in arts and sports, alleviate academic pressure, and promote holistic competencies (Bray, 2012). This study’s findings validate these goals: academic tutoring shows no substantive association with improvements in academic achievement, excessive academic tutoring time negatively impacts artistic development and well-being, and the number of tutoring programs attended has limited impact. Meaningful engagement in sports and arts tutoring proves beneficial, supporting the evidence base for current governance efforts.

Fourth, greater support should be provided to students from economically and culturally disadvantaged backgrounds to promote educational equity. Out-of-school tutoring, as privatized supplementary education, disproportionately benefits higher-status families, exacerbating inequality. This study shows students from families with greater economic and cultural capital have access to more diverse and higher-quality tutoring. Recognizing tutoring’s reproductive role highlights the need to address these disparities.

Support for disadvantaged students should focus on expanding access to arts and sports programs. Schools play a critical role; for example, following the “Double Reduction” policy in China (Xue & Li, 2023), many have integrated more arts and physical education into after-school services. Policy interventions should also promote reasonable pricing, reduce costs of arts and sports tutoring, and improve accessibility for lower-income families. Such measures are essential to narrow educational opportunity gaps and foster inclusive, equitable learning environments.

## 7. Conclusions

Compared with existing studies on compensatory education that primarily focus on the effects of academic tutoring on academic achievement, this study adopts a more comprehensive and nuanced approach by drawing on the theory of student engagement. By distinguishing academic, arts, and sports tutoring and unpacking investment in terms of time, engagement, and intensity, the study highlights the differentiated roles that tutoring plays across multiple developmental domains.

First, the effectiveness of different types of out-of-school tutoring is clearly differentiated. Total investment in academic tutoring shows no significant association with academic achievement, and total investment in arts programs is not significantly related to artistic literacy, whereas investment in sports programs is significantly and positively associated with students’ physical and mental health. Second, marked differences emerge across types of investment. Neither the number of tutoring programs nor financial expenditure across all types of out-of-school tutoring is significantly associated with holistic competence. In contrast, time investment in sports programs is positively related to social practice competence and physical and mental health, while time investment in academic tutoring is negatively associated with artistic literacy and physical and mental health. Moreover, cognitive investment in sports programs and emotional investment in arts programs are significantly and positively associated with social practice competence and moral character, respectively. Other associations involving behavioral, cognitive, and emotional engagement are not statistically significant. In addition, no type of out-of-school tutoring investment is significantly associated with academic achievement.

From a policy perspective, the results imply that the reproductive function of compensatory education through academic tutoring may be limited. Managing academic burden while encouraging balanced participation in sports and arts programs may better support holistic human development. In this sense, the study provides empirical grounding for governance approaches that restrict excessive academic tutoring while offering guided support for non-academic enrichment.

This study also has several limitations. The first limitation largely relates to constraints associated with holistic competence assessment. Although this study relies on high-stakes assessment results administered by educational authorities to substantiate the validity of the holistic competence measure, no additional sources of validity evidence were provided. The sample was limited to a single provincial capital city in central China, which restricts the generalizability of the findings. Notably, this city has implemented a holistic competence evaluation system for over two decades, and the results have long served as an important criterion for student progression, which partially justifies its use in this study. However, contextual factors may differ substantially across regions. For example, the degree of marketization of tutoring services, the intensity of policy enforcement under the Double Reduction reform, and local assessment practices related to holistic competence evaluation may vary across provinces and school systems. These differences may shape both students’ tutoring participation patterns and the interpretation of holistic competence outcomes. Second, students retrospectively reported their participation in out-of-school tutoring after entering Grade 10, which may introduce recall bias and affect data accuracy. While recall bias cannot be entirely eliminated, structured learning activities such as out-of-school tutoring are inherently less prone to recall errors than transient or episodic interactions. To further mitigate the potential impact of recall inaccuracies, this study measured tutoring participation intensity using ordinal categorical variables rather than precise numerical values. It should be noted that detail omissions stemming from faded memories, as well as socially desirable response bias potentially induced by the Double Reduction policy, may have led to the underreporting of actual tutoring participation. This implies that the statistical correlations identified in the present study are likely more conservative than the true relationships in practice. Third, the study is subject to the issue of statistical multiplicity, given that multiple regression models were estimated to examine the associations between different tutoring types and various outcome variables of holistic competence. Although bootstrap resampling techniques were employed to enhance the robustness of statistical inferences, the risk of false-positive results arising from multiple comparisons cannot be fully ruled out. For this reason, readers are advised to prioritize the magnitude of effect sizes and cross-model comparative patterns when interpreting the study’s findings, rather than overinterpreting isolated statistically significant *p*-values. In addition, holistic competence ratings may exhibit restricted variance or potential ceiling effects, as students’ scores in the official assessment system tend to cluster toward higher categories. This limited variability may partly explain why some tutoring forms show weak or null effects in certain models.

Future research will seek more robust and innovative methods for assessing holistic competence, expand the geographic scope of the sample to enhance generalizability, and apply more sophisticated analytical models to explore the complex pathways linking different types of out-of-school tutoring investment to holistic competence. Such efforts are expected to yield deeper insights into the roles and functions of compensatory education.

## Figures and Tables

**Table 1:** Descriptive Statistics of Out-of-School Tutoring Engagement and Holistic Competencies.

Variable	Min	Max	Mean	Standard Deviation	Sample Size
Number of Sports Programs	1	10	2.56	1.71	391
Financial Investment in Sports (1–5)	1	5	2.27	1.05	391
Time Investment in Sports (1–7)	1	7	2.51	1.52	391
Number of Arts Programs	1	18	2.65	2.04	485
Financial Investment in Arts (1–5)	1	5	2.76	1.13	485
Time Investment in Arts (1–7)	1	7	2.43	1.02	485
Number of Academic Programs	1	10	5.23	2.16	640
Financial Investment in Academics (1–5)	1	5	3.86	1.25	640
Time Investment in Academics (1–6)	1	6	4.02	1.15	640
Moral Character (1–4)	1	4	3.93	0.33	704
Academic Performance (1–4)	1	4	3.67	0.59	704
Social Practice (1–4)	1	4	3.77	0.50	704
Artistic Literacy (1–4)	1	4	3.81	0.49	704
Physical and Mental Health (1–4)	1	4	3.74	0.57	704

**Table 2:** Effects of Overall Tutoring Engagement on Holistic Competencies.

Predictors	Moral Character	Academic Performance	Social Practice	Artistic Literacy	Physical and Mental Health
Gender	0.01	0.11 **	0.05	−0.08 *	0.06
Household registration	0.03	−0.02	0.01	−0.04	−0.07
School Type	0.08 *	0.18 ***	0.11 *	0.13 *	0.06
Father’s education	−0.04	0.12 *	0.06	0.09	0.03
Mother’s education	0.01	−0.03	0.03	−0.07	−0.01
Household income	0.09 *	0.10 *	0.15 **	0.10 *	0.07
Primary School Ranking	0.09 *	0.18 ***	0.12 *	0.10 *	0.10 *
Place of primary school	0.01	0.02	0.01	0.08 *	0.10 *
Place of Junior Secondary School	0.06	0.06	0.06	0.08 *	0.05
Sports Tutoring Engagement	0	−0.05	0.04	−0.02	0.17 **
Arts Tutoring Engagement,	−0.01	0.05	−0.01	0.11 *	−0.07
Academic Tutoring Engagement	0.07	0	0.05	0.06	0.04
R^2^	0.05	0.12	0.1	0.1	0.09
F	2.59 **	7.61 ***	6.26 ***	6.19 ***	5.15 ***

Note: All effects are standardized beta coefficients from a multiple regression. * *p* < 0.05, ** *p* < 0.01, *** *p* < 0.001.

**Table 3:** Effects of Engagement Dimensions by Tutoring Type on Holistic Competencies.

Tutoring Type & Dimensions	Moral Character	Academic Performance	Social Practice	Artistic Literacy	Physical and Mental Health
	Sports Tutoring				
Amount	−0.04	0.06	0.06	−0.07	0
Money	0.02	−0.06	−0.08	−0.06	−0.08
Time	0.09	0.02	0.13 *	0.11	0.14 *
Behavior	0.05	0.3	0.2	0.24	0.31
Cognitive	0.25	−0.24	0.23 *	−0.02	−0.23
Affective	−0.34	−0.07	−0.45	−0.24	−0.03
	Arts Tutoring				
Amount	0	0	−0.03	−0.02	−0.06
Money	0	−0.05	−0.09	0.12	−0.09
Time	−0.15	−0.04	0.01	−0.02	0.04
Behavior	−0.4	0.3	−0.05	−0.15	0.08
Cognitive	0.01	−0.18	0.09	0.2	−0.22
Affective	0.52 *	−0.03	0.02	0.06	0.25
	Academic Tutoring				
Amount	−0.02	−0.05	0	−0.02	0.04
Money	0.14 *	−0.03	0.07	0.01	0.01
Time	−0.07	−0.07	−0.08	−0.14 **	−0.19 ***
Behavior	0.1	0.18	0.23	−0.03	0.18
Cognitive	−0.05	0.03	0.05	0.21	−0.03
Affective	−0.01	−0.07	−0.2	0	−0.02

Note: All effects are standardized beta coefficients from a multiple regression. * *p* < 0.05, ** *p* < 0.01, *** *p* < 0.001.

## Data Availability

The data presented in this study are available on request from the corresponding author. The data are not publicly available due to privacy and ethical restrictions, as the dataset contains sensitive information regarding student performance and educational assessments.

## Appendix A

**Table A1:** Variables and Measurement Methods.

Variable Name	Definition and Measurement
Covariates	
Gender	Student gender (Female = 0, Male = 1)
Type of Household Registration	Type of household registration (Rural = 0, Urban = 1)
Type of school	Type of junior secondary school attended (Non-key school = 0, Key school = 1)
Father’s Education Level	Educational attainment (Middle school or below = 0, High school = 1, Associate degree = 2, Bachelor’s degree = 3, Graduate degree = 4)
Mather’s Education Level	Educational attainment (Middle school or below = 0, High school = 1, Associate degree = 2, Bachelor’s degree = 3, Graduate degree = 4)
Annual Household Income	(Below ¥50,000 = 1; ¥50,000–¥99,999 = 2; ¥100,000–¥199,999 = 3; ¥200,000–¥299,999 = 4; Above ¥300,000 = 5)
Academic Ranking	Approximate class rank at primary school graduation (Top 5% = 1; 5–10% = 2; 10–30% = 3; 30–50% = 4; 50–70% = 5; Below 70% = 6)
Primary School Location	Whether student attended primary school in local city (No = 0, Yes = 1)
Junior Secondary School Location	Whether student attended junior secondary school in local city (No = 0, Yes = 1)
Independent Variables (for academic, arts, and sports tutoring separately)	
Number of Programs Participated	Count of out-of-school tutoring programs in each category
Monthly Cost	Average monthly spending per category (Below ¥500 = 1; ¥501–1000 = 2; ¥1001–2000 = 3; ¥2001–3000 = 4; Above ¥3000 = 5)
Weekly Time Investment	Weekly hours invested in each tutoring type (Below 1 h = 1; 1–3 h = 2; 3–5 h = 3; 5–7 h = 4; 7–9 h = 5; 9–11 h = 6; Over 11 h = 7)
Behavioral Engagement	Measured by punctual attendance, task completion, participation in class, and interaction with teachers and peers
Cognitive Engagement	Measured by awareness of content relevance, alignment with personal needs, self-regulation during learning, and improvement of learning strategies
Affective Engagement	Measured by autonomy in choice, learning motivation, self-confidence, satisfaction with tutoring, and enjoyment of learning experience
Dependent Variables	
Moral Character, Academic Performance, Social Practice, Artistic Literacy, Physical and Mental Health	Holistic competencies (5 Dimensions), derived from the official assessment results. Each rated on a 4-level scale: D = 1, C = 2, B = 3, A = 4.

## Appendix B

**Table A2:** Group Differences in Tutoring Engagement Intensity.

Subgroups	Sports			Arts			Academic		
	Amount	Money	Time	Amount	Money	Time	Amount	Money	Time
GEN (F-value)	5.81 *	0.00	0.37	16.16 ***	1.72	3.13	0.90	0.69	0.22
Female (1)	0.96 (1.55)	0.94 (1.32)	1.01 (1.57)	2.39 (2.07)	2.33 (1.50)	2.07 (1.37)	4.70 (2.45)	3.45 (1.62)	2.01 (1.27)
Male (2)	1.82 (1.91)	1.50 (1.35)	1.69 (1.71)	1.21 (1.59)	1.46 (1.55)	1.27 (1.34)	4.74 (2.69)	3.45 (1.67)	2.00 (1.28)
HUR (F-value)	3.96 *	0.18	5.71 *	2.69	0.01	1.85	24.90 ***	11.42 **	0.01
Rural	1.10 (1.76)	1.05 (1.34)	1.33 (1.87)	1.21 (1.71)	1.46 (1.62)	1.33 (1.53)	4.06 (2.46)	3.13 (1.75)	1.93 (1.37)
Urban	1.57 (1.81)	1.32 (1.37)	1.40 (1.57)	2.09 (1.97)	2.10 (1.52)	1.83 (1.31)	5.09 (2.57)	3.62 (1.57)	2.04 (1.22)
SCT (F-value)	13.03 ***	0.88	1.06	8.07 **	2.66	0.53	21.72 ***	7.73 **	5.81 *
Non-key school	1.10 (1.53)	1.11 (1.37)	1.25 (1.67)	1.51 (1.78)	1.70 (1.57)	1.52 (1.37)	4.27 (2.46)	3.23 (1.73)	1.85 (1.32)
Key school	1.78 (2.01)	1.38 (1.34)	1.50 (1.67)	2.14 (2.01)	2.11 (1.59)	1.83 (1.41)	5.32 (2.61)	3.72 (1.51)	2.19 (1.18)
MED (F-value)	4.90 **	1.75	0.59	5.48 ***	4.04 **	2.29	5.53 ***	2.66 **	4.65 **
Middle school (below)	1.16 (1.60)	1.08 (1.35)	1.24 (1.69)	1.22 (1.71)	1.37 (1.46)	1.24 (1.37)	4.19 (2.65)	3.12 (1.79)	1.89 (1.47)
High school	1.06 (1.61)	1.00 (1.26)	1.25 (1.71)	1.35 (1.69)	1.61 (1.59)	1.41 (1.38)	4.46 (2.32)	3.41 (1.59)	1.96 (1.11)
Associate degree	1.49 (1.86)	1.23 (1.33)	1.36 (1.65)	2.02 (1.91)	2.06 (1.49)	1.93 (1.45)	4.82 (2.63)	3.49 (1.69)	1.93 (1.15)
Bachelor’s degree	1.75 (1.79)	1.55 (1.45)	1.54 (1.59)	2.29 (1.98)	2.30 (1.58)	1.91 (1.25)	5.29 (2.54)	3.75 (1.46)	2.11 (1.21)
Graduate degree	2.77 (2.83)	1.77 (1.42)	2.00 (2.14)	3.54 (2.49)	3.15 (1.52)	2.65 (1.60)	5.69 (2.75)	3.73 (1.61)	2.92 (1.88)
FED (F-value)	8.32 ***	2.37	0.43	1.89	0.93	1.27	3.71 **	3.34 *	0.28
Middle school (below)	1.16 (1.66)	1.08 (1.35)	1.26 (1.68)	1.46 (1.93)	1.70 (1.65)	1.50 (1.50)	4.65 (2.72)	3.41 (1.73)	1.98 (1.33)
High school	1.08 (1.56)	0.99 (1.23)	1.29 (1.78)	1.32 (1.71)	1.34 (1.52)	1.19 (1.31)	4.22 (2.37)	3.16 (1.67)	1.93 (1.33)
Associate degree	1.47 (1.74)	1.35 (1.41)	1.38 (1.61)	1.98 (2.16)	2.04 (1.58)	1.85 (1.45)	4.82 (2.47)	3.61 (1.58)	2.07 (1.13)
Bachelor’s degree	1.55 (1.77)	1.35 (1.40)	1.45 (1.65)	2.06 (1.67)	2.19 (1.46)	1.89 (1.25)	5.01 (2.68)	3.58 (1.60)	2.05 (1.26)
Graduate degree	2.81 (2.72)	1.76 (1.46)	1.66 (1.68)	2.86 (2.37)	2.71 (1.50)	2.40 (1.53)	5.26 (2.49)	3.67 (1.59)	2.00 (1.47)
HIN (F-value)	4.98 **	4.12 **	0.35	9.04 ***	4.75 **	1.23	7.31 ***	7.19 ***	5.67 ***
Below ¥50,000	0.86 (1.38)	0.85 (1.20)	1.06 (1.72)	1.36 (1.64)	1.68 (1.59)	1.56 (1.43)	4.03 (2.78)	2.99 (1.92)	1.81 (1.37)
¥50,000–¥99,999	1.12 (1.69)	0.95 (1.30)	1.14 (1.65)	1.60 (1.62)	1.87 (1.48)	1.66 (1.40)	4.14 (2.60)	3.07 (1.73)	1.74 (1.17)
¥100,000–¥199,999	1.22 (1.57)	1.12 (1.26)	1.36 (1.70)	1.54 (1.64)	1.65 (1.46)	1.51 (1.28)	4.56 (2.45)	3.36 (1.60)	1.90 (1.19)
¥200,000–¥299,999	1.84 (1.98)	1.59 (1.41)	1.57 (1.62)	2.12 (2.02)	2.16 (1.63)	1.78 (1.35)	5.53 (2.41)	4.03 (1.37)	2.24 (1.21)
Above ¥300,000	2.39 (2.21)	1.94 (1.50)	1.86 (1.70)	2.59 (2.68)	2.28 (1.82)	1.93 (1.69)	5.57 (2.61)	3.85 (1.54)	2.45 (1.46)
PSR (F-value)	1.03	0.48	1.97	1.83	1.16	0.29	0.78	0.92	1.19
Top 5%	1.47 (1.90)	1.23 (1.37)	1.39 (1.70)	1.91 (2.08)	1.91 (1.66)	1.65 (1.42)	4.67 (2.60)	3.49 (1.66)	2.07 (1.36)
5–10%	1.28 (1.76)	1.09 (1.34)	1.06 (1.31)	1.88 (1.96)	1.93 (1.41)	1.75 (1.36)	4.72 (2.49)	3.52 (1.60)	2.01 (1.23)
10–30%	1.20 (1.52)	1.21 (1.32)	1.43 (1.76)	1.45 (1.59)	1.74 (1.58)	1.58 (1.46)	4.83 (2.41)	3.43 (1.52)	1.94 (0.98)
30–50%	1.77 (1.79)	1.58 (1.33)	1.94 (2.01)	1.64 (1.68)	1.84 (1.56)	1.70 (1.40)	4.98 (2.75)	3.34 (1.74)	1.95 (1.33)
50–70%	1.75 (2.07)	1.29 (1.41)	1.29 (1.86)	1.43 (1.55)	1.86 (1.63)	1.46 (1.23)	4.61 (3.00)	3.00 (1.85)	1.64 (1.23)
Below 70%	1.16 (1.74)	1.32 (1.73)	1.68 (2.26)	1.32 (1.92)	1.42 (2.01)	1.16 (1.80)	4.00 (2.77)	3.32 (2.06)	2.05 (1.78)
PPS (F-value)	1.42	0.94	1.91	2.62 **	−0.18	−0.13	0.86	0.96	0.45
Not in local city	1.58 (2.09)	1.32 (1.44)	1.58 (1.94)	1.91 (2.36)	1.73 (1.62)	1.52 (1.53)	4.83 (2.86)	3.52 (1.70)	2.03 (1.35)
In local city	1.37 (1.74)	1.21 (1.35)	1.33 (1.63)	1.75 (1.84)	1.90 (1.58)	1.68 (1.39)	4.70 (2.53)	3.44 (1.64)	2.00 (1.26)

Note: GEN—Gender, HUR—Household registration, SCT—School Type, FED—Father’s education, MED—Mother’s education, HIN—Household income, PSR—Primary School Ranking, PPS—Place of primary school. * *p* < 0.05, ** *p* < 0.01, *** *p* < 0.001.

## Appendix C

Questionnaire on Engagement in Out-of-School Tutoring
Engagement Items (5-point Likert Scale):

Please indicate your level of agreement with the following statements: 1 = Strongly disagree; 2 = Disagree; 3 = Neutral; 4 = Agree; 5 = Strongly agree.

Behavior Engagement

1. During competitions, I cooperate closely with my teammates in order to win.
2. In team activities, I actively communicate with others and try my best to complete tasks.
3. I attend sports tutoring on time and never arrive late.
4. I take the tutoring very seriously, and I feel a sense of urgency if I do not complete the tasks.
5. During training, I pay attention to my progress compared with others and push myself to complete tasks on time.

Cognitive Engagement

1. Before participating, I collected relevant information and had a detailed understanding of the training content and procedures.
2. Before training, I realized the importance of sports tutoring for my physical fitness and health literacy.
3. I have learned in detail about the teacher’s teaching style and the curriculum arrangement of the program.
4. During tutoring, I learn from others’ strengths and make up for my weaknesses.
5. After training, I report my task completion to the teacher and make improvements.
6. I communicate with peers and exchange problems encountered during tutoring in order to make progress together.

Affective Engagement

1. Participating in sports tutoring makes me happy.
2. Whenever I complete a training task, I feel more confident.
3. During tutoring, I get along well with teachers and classmates.
4. Classmates’ enthusiasm and teachers’ passion motivate me during training.
5. Participating in sports tutoring is my own choice, not required by parents or teachers.
6. Learning new skills and exercising makes me feel happy and fulfilled.
